# Changing drinking patterns among Italians: 7 out of 10 students experience Binge Drinking

**DOI:** 10.1192/j.eurpsy.2024.306

**Published:** 2024-08-27

**Authors:** F. Marcolini, D. De Ronchi, A. R. Atti

**Affiliations:** Department of Biomedical and Neuromotor Sciences, University of Bologna, Bologna, Italy

## Abstract

**Introduction:**

The expression Binge Drinking (BD) refers to dysregulated alcohol consumption, characterized by the intake of large quantities of alcohol, regardless of their nature, consecutively in a limited period of time. BD is a significant public health problem in many European countries, including Italy. According to data from the *Istituto Superiore di Sanità*, dated 2020, over 4 million Italians exhibit episodic excessive alcohol consumption (compared to 2019 data, there was an increase of approximately 5,3%).

**Objectives:**

This study aims to examine alcohol consumption habits in the Italian population, evaluating psychopathological correlations that can explain its diffusion.

**Methods:**

Between January and May 2023, an anonymous online questionnaire was randomly sent to the general population. Alongside with tests to evaluate psycho-social features, to estimate the presence of alcohol abuse or dependence the AUDIT scale (Saunders *et al.* Addict Abingdon Engl. 1993; 88:791–804) was used. It included two specific questions to frame the phenomenon of BD (Cranford *et al*. Alcohol Clin Exp Res. 2006; 30:1896–905). No other study conducted in Italy has so far used the aforementioned validated questions.

**Results:**

The sample consists of 308 people (189 F, 119 M), with an average age of 32 years (*sd* 14). The AUDIT indicates a state of chronic alcohol consumption in 11,7% (95% confidence interval 8,5%-15,7%), of the recruited sample, positively correlating with the element of impulsivity (*p*<0,005) confirming what has already been reported in literature. BD prevalence reaches 56% (M 57%, F 55%) without any significant correlation with impulsivity, personality disorders, emotional dysregulation, or sensitivity to rejection. Among university students the prevalence of BD exceeds 70% (95% confidence interval 60%-76%), with a number of drinks reported for a single occasion reaching up to 25 units and a reported number of binge episodes, in a two-week span, ranging from 2 to 10.

**Conclusions:**

Despite possible *biases,* this study raises the relevant issue of the extremely high prevalence of BD disorder, which is particularly alarming in light of the numerous issues related to the behavior itself. A direct correlation with reduced school performance, an increase in risky sexual behavior, and an increase in cases of drunk driving have been evaluated. Considering these consequences, it is of primary importance on a medical, but even more social level, to best characterize this phenomenon in such a way as to be able to implement awareness-raising and prevention interventions.

**Disclosure of Interest:**

None Declared

